# Estimating Fisher discriminant error in a linear integrator model of neural population activity

**DOI:** 10.1186/s13408-021-00104-4

**Published:** 2021-02-19

**Authors:** Matias Calderini, Jean-Philippe Thivierge

**Affiliations:** 1 School of Psychology, University of Ottawa, 136 Jean Jacques Lussier, Ottawa, ON K1N 6N5 Canada; 2 Brain and Mind Research Institute, University of Ottawa, Ottawa, ON K1N 6N5 Canada

**Keywords:** Linear model, Fisher linear discriminant analysis, Noise correlation

## Abstract

Decoding approaches provide a useful means of estimating the information contained in neuronal circuits. In this work, we analyze the expected classification error of a decoder based on Fisher linear discriminant analysis. We provide expressions that relate decoding error to the specific parameters of a population model that performs linear integration of sensory input. Results show conditions that lead to beneficial and detrimental effects of noise correlation on decoding. Further, the proposed framework sheds light on the contribution of neuronal noise, highlighting cases where, counter-intuitively, increased noise may lead to improved decoding performance. Finally, we examined the impact of dynamical parameters, including neuronal leak and integration time constant, on decoding. Overall, this work presents a fruitful approach to the study of decoding using a comprehensive theoretical framework that merges dynamical parameters with estimates of readout error.

## Introduction

In recent years, neuronal decoding has emerged as a key aspect of understanding the neural code [1]. The aim of decoding algorithms is to read out the sensory-driven responses of a neuronal population and classify them following a given criterion. Popular criteria include Fisher information [2, 3], mutual information [4], and machine learning approaches [5, 6]. While many types of decoders exist [7], a linear readout of neural activity has often been employed to perform sensory classification [8, 9] and predict motor decisions [10, 11]. Further, different classes of linear readouts are amenable to mathematical analysis and capture biological learning rules such as Hebbian learning [12].

In this work, we formally analyze the optimal decoding error of a linear decoder based on Fisher linear discriminant analysis (LDA). Assuming discrete classes of stimuli, LDA provides an upper bound on linear decoding capacity [13]. In addition, LDA shows good agreement with decision-making tasks and offers a bridge between cortical activity and behavioral performance [14, 15].

Importantly, most theoretical approaches based on neural decoding are not concerned with how linear decoders would be influenced by specific dynamical parameters of modeled neural systems [16]. Here, we address this concern by providing expressions that relate decoding error to the adjustable parameters of a rate-based population model with a linear neural integrator [17, 18]. This model captures the average spiking activity of neuronal populations [19–21] and the quasi-linear responses of neurons found in many experimental contexts [22]. Preliminary results have been presented in previous work [13, 14], yet the full analytical solution had remained incomplete and limited to positive noise correlation; we now present the complete solution.

The framework relies on the simplifying assumption that signal and noise correlations originate from independent sources. While this assumption does not hold in biological circuits, where signal and noise are related [1], it allows us to systematically explore a wide range of scenarios that describe the impact of neuronal inputs, noise, correlations, and dynamical parameters on linear decoding, where the contribution of each parameter can be examined independently.

This paper begins by describing the neural integrator model and the LDA readout. Then, we provide expressions for LDA error that rely on the parameters of the integrator model. Finally, we consider the effect of correlation, noise, and dynamical parameters on neuronal decoding using both analytical expressions and numerical simulations.

## Linear population model

**Figure 1:**
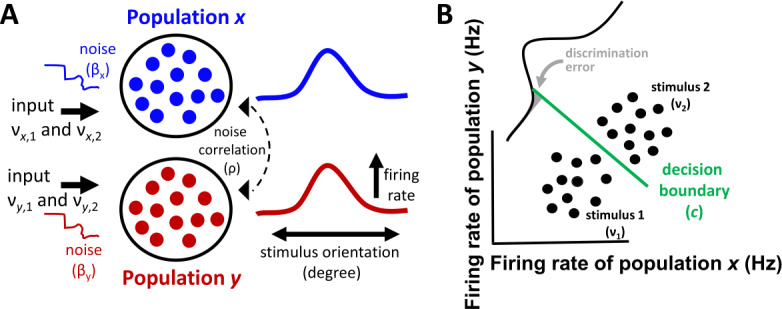
Fisher linear discrimination of neural activity in a population model. (**A**) Two neural populations (*x* and *y*) where the noise correlation is adjusted via a parameter *ρ*. Each population receives two distinct inputs ($\nu _{1}$ and $\nu _{2}$) and a private source of noise whose gain is $\beta _{\mathrm{x}}$ and $\beta _{\mathrm{y}}$, respectively. The stimulus-driven response of each population is described by a tuning curve relating stimulus orientation to firing rate. (**B**) Activity for populations *x* and *y* is shown at discrete time-points (solid black circles). The optimal decision boundary (*c*) obtained by LDA discriminates amongst the neural activity generated by each of the two inputs. Neural responses follow a Gaussian distribution. The shaded area shows the proportion of discrimination error for stimulus 2

As a starting point, we assume two independent neuronal populations, each projecting in a feedforward manner to a readout discriminating amongst two inputs, $\nu _{1}$ and $\nu _{2}$, that are constant over time (Fig. 1(A)). Each population’s mean firing rate in response to stimuli is conceptualized by a tuning curve where a stimulus feature, for instance visual orientation, generates a graded response. This scenario is analogous to analyses that examine population responses after performing a dimensionality reduction to generate a “population tuning curve” [23]. While a more complex model could account for a heterogeneity of responses within each population, we choose to limit our model to two homogeneous populations in order for the classification problem to remain tractable.

The activity of each population is described by a linear neural integrator

1 $$\begin{aligned} &\tau _{x} \frac{d x_{i}}{dt} =- \alpha _{x} x_{i} + \nu _{i,x} + \beta _{x} \xi _{x} (t),\\ & \tau _{y} \frac{d y_{i}}{dt} =- \alpha _{y} y_{i} + \nu _{i,y} + \beta _{y} \xi _{y} ( t ), \end{aligned}$$

where $x_{i}$ and $y_{i}$ are the firing rates of each population in response to a given stimulus *i*, *τ* is a time constant, *α* is a leak term, $\xi (t)$ is Gaussian white noise ($\mathcal{N} (0,1)$), and *β* is the gain of the noise. Network parameters *τ*, *α*, and *β* are bound to $\mathbb{R}_{>0}$. We make no distinction between noise induced by stimuli and noise generated through intrinsic neural activity. While their effect on mean rate activity is similar [24], their impact on noise correlations differs [1]; in the model, we explicitly separate the effect of firing rate and noise correlation. This will be done by controlling noise correlation through a tunable parameter, as detailed in Sect. 4. An advantage of this formalism is that the effect of noise correlation can be systematically isolated from changes in firing rates and signal correlation that would be induced through reciprocal connections between the two populations. Further, depending on the choice of parameters, the addition of recurrent weights to Eq. (1) may prevent the system from reaching a stationary state, which is a fundamental assumption of LDA.

## Fisher linear discriminant decoder

A linear decoder based on LDA reads out the activity of the population model in order to perform a binary discrimination (Fig. 1(B)). Discrimination error generated by LDA provides an estimate of the statistical confidence in distinguishing pairs of stimuli based on network activity. We focus on pairwise discrimination given that error rates obtained from more than two stimuli are well approximated by values obtained from all combinations of pairwise comparisons [25].

LDA assumes that neural activity is sampled from a multivariate Gaussian distribution with class covariance matrix $\Sigma _{i}$ and class mean vector $\boldsymbol{\mu }_{i}$. Further, LDA assumes equal class covariance, therefore $\Sigma _{1} = \Sigma _{2} = \Sigma $. LDA attempts to find a projection line *w*, perpendicular to the decision boundary, onto which the input space is projected. The optimal projection line maximizes the Fisher criterion *J* (*w*) defined as the ratio of the projected between- to within-class variance:

$$ J ( w ) = \frac{w\boldsymbol{\cdot }( \boldsymbol{\mu }_{2} - \boldsymbol{\mu }_{1} )^{2}}{w^{T} \boldsymbol{\cdot } \Sigma _{W} \boldsymbol{\cdot }w}. $$

Given the assumption of equal class covariance, we set $\Sigma _{W} =2 \Sigma $. By taking the derivative of *J* (*w*) with respect to *w* and setting it to zero, one finds the closed-form solution for the optimal projection line to be

2 $$ W= ( 2 \Sigma )^{-1} ( \boldsymbol{\mu }_{2} - \boldsymbol{ \mu }_{1} ). $$

## Formulating a model-based linear decoder

To analytically derive means ($\boldsymbol{\mu }_{1}$ and $\boldsymbol{\mu }_{2}$) and covariance (Σ) from the neural population model, we rearrange Eq. (1) as follows, using population *x* as example:

3 $$ d x_{i} = \frac{\alpha _{x}}{\tau _{x}} \biggl( \frac{\nu _{ix}}{\alpha _{x}} - x_{i} \biggr) \,dt+ \frac{\beta _{x}}{\tau _{x}} \xi _{x} ( t ) \,dt. $$

Given that a white noise process is by definition the time derivative of a Weiner process, $\xi ( t ) =d W_{t} /dt$, we can rewrite Eq. (3) as

4 $$ d x_{i} = \theta _{x} ( \mu _{ix} - x_{i} ) \,dt+ \lambda _{x} \,d W_{x,t}, $$

with $\theta _{x} = \alpha _{x} / \tau _{x}$, $\mu _{ix} = {\nu _{ix}}/{\alpha _{x}}$, and $\lambda _{x} = \beta _{x} / \tau _{x}$. Equation (4) is an Orstein–Uhlenbeck process with known solution

5 $$ x_{i} ( t ) = \mu _{ix} + ( x_{i0} - \mu _{ix} ) e^{- \theta _{x} t} +\lambda \int _{0}^{t} e^{- \theta _{x} ( t-s )} \,dB ( s ). $$

Equation (5) is a mean reverting process whose stable state follows a Gaussian distribution. A full derivation of this process is found in Sections A.1–A.2. To summarize this derivation, the expected mean and variance are

$$\begin{aligned} &E \bigl[ x_{i} ( t ) \bigr] = \mu _{ix} + ( x_{i0} - \mu _{ix} ) e^{- \theta _{x} t}, \\ &\operatorname{Var} \bigl( x_{i} ( t ) \bigr) = \frac{\lambda _{x}^{2}}{2 \theta _{x}} \bigl( 1- e^{- 2\theta _{x} t} \bigr). \end{aligned}$$

The stationary mean and variance of Eq. (5) are

$$\begin{aligned} &\lim_{t\rightarrow \infty } E [ x_{i} ] = \mu _{ix} = \frac{\nu _{x,i}}{\alpha _{x}}, \\ &\lim_{t\rightarrow \infty } \operatorname{Var} ( x_{i} ) = \frac{\lambda _{x}^{2}}{2 \theta _{x}} = \frac{\beta _{x}^{2}}{2 \tau _{x} \alpha _{x}} = \sigma ^{2}. \end{aligned}$$

With the assumption that the mean of *x* is much larger than the variance, there is negligible probability that *x* would fall below zero. Imposing strictly positive values of *x* could be achieved by the addition of a constant and would not alter the results obtained from the linear classifier.

The readout of neural activity depends on the following feature space:

$$\begin{aligned} &Z\sim \mathcal{N} ( \boldsymbol{\mu }_{i}, \boldsymbol{\Sigma } ), \\ &\boldsymbol{\mu }_{i} = [ \mu _{xi}, \mu _{yi} ]^{T}, \\ &\boldsymbol{\Sigma } = \begin{bmatrix} \sigma _{x}^{2} & \rho \sigma _{x} \sigma _{y}\\ \rho \sigma _{x} \sigma _{y} & \sigma _{y}^{2} \end{bmatrix}, \end{aligned}$$

where *Z* is obtained from the probability distribution of a multivariate Gaussian with mean $\boldsymbol{\mu }_{i}$ and covariance **Σ**. Setting the parameter $\rho =0$ would be equivalent to a so-called “diagonal decoder” where off-diagonal elements of the covariance matrix are neglected, thus ignoring noise correlations altogether [16].

The closed form solution of LDA (Eq. (2)) can be expressed using the parameters of the population model (Eq. (1)) as follows. First, the total within-class scatter $S_{w}$ is

$$\begin{aligned} S_{w} &=2 \Sigma \\ &=2 \begin{bmatrix} \sigma _{x}^{2} & \rho \sigma _{x} \sigma _{y}\\ \rho \sigma _{x} \sigma _{y} & \sigma _{y}^{2} \end{bmatrix} \\ &=2 \begin{bmatrix} \frac{\beta _{x}^{2}}{2 \tau _{x} \alpha _{x}} & \rho \sqrt{\frac{\beta _{x}^{2}}{2 \tau _{x} \alpha _{x}} \frac{\beta _{y}^{2}}{2 \tau _{y} \alpha _{y}}}\\ \rho \sqrt{\frac{\beta _{x}^{2}}{2 \tau _{x} \alpha _{x}} \frac{\beta _{y}^{2}}{2 \tau _{y} \alpha _{y}}} & \frac{\beta _{y}^{2}}{2 \tau _{y} \alpha _{y}} \end{bmatrix}. \end{aligned}$$

To alleviate the notation, we define $\Delta \boldsymbol{\mu }^{T} = [ \Delta \mu _{x}, \Delta \mu _{y} ]^{T}= \boldsymbol{\mu }_{0} - \boldsymbol{\mu }_{1}$, where $\Delta \mu _{u} = \Delta \nu _{u} / \alpha _{u}$, and $\Delta \nu _{u}$ is the absolute difference between the two stimuli, given an index *u* that stands for either population *x* or *y*. In this way, Eq. (2) becomes

$$\begin{aligned} W&= (2 \Sigma )^{-1} \Delta \boldsymbol{\mu } \\ &= \frac{1}{2(1- \rho ^{2} )} \begin{bmatrix} \frac{1}{\sigma _{x}^{2}} \Delta \mu _{x} -\rho \frac{1}{\sqrt{\sigma _{x}^{2} \sigma _{y}^{2}}} \Delta \mu _{y}\\ \frac{1}{\sigma _{y}^{2}} \Delta \mu _{y} -\rho \frac{1}{\sqrt{\sigma _{x}^{2} \sigma _{y}^{2}}} \Delta \mu _{x} \end{bmatrix} \\ &= \frac{1}{2(1- \rho ^{2} )} \begin{bmatrix} \frac{\tau _{x}}{\beta _{x}^{2}} \Delta \nu _{x} -\rho \sqrt{\frac{\tau _{x} \alpha _{x}}{\beta _{x}^{2}} \frac{\tau _{y}}{\beta _{y}^{2} \alpha _{y}}} \Delta \nu _{y}\\ \frac{\tau _{y}}{\beta _{y}^{2}} \Delta \nu _{y} -\rho \sqrt{\frac{\tau _{y} \alpha _{y}}{\beta _{y}^{2}} \frac{\tau _{x}}{\beta _{x}^{2} \alpha _{x}}} \Delta \nu _{x} \end{bmatrix}. \end{aligned}$$

From the law of total probability, the error rate of classification is given by

6 $$ \varepsilon =P [ y=0 \vert k=1 ] P [ k=1 ] +P [ y=1 \vert k=0 ] P [ k=0 ], $$

where $P [ k=1 ]$ is the probability that a randomly sampled point from any distribution belongs to class *j* and $P [ y=i \vert k=j ]$ is the probability that a point is classified as belonging to class *i* when it belongs to class *j*. Given that the classifier is unbiased towards each of the two neural populations, $P [ k=0 ] =P [ k=1 ] =0.5$. To calculate conditional probabilities $P [ y=i \vert k=j ]$, one must define a threshold *c* that serves as a boundary between the two distributions. The value of *c* is chosen to be the midpoint between the means of the projected distributions.

We calculate $P [ y=i \vert k=j ]$ as the area under the curve of the density function for *j* in the region where *i* is the correct class. As a first step, we shift the projected distributions by a factor *c*, so that the threshold becomes zero to simplify the integration. More specifically, the unshifted threshold *c*, the means of the shifted distributions $\eta _{i}$, and their variance $\zeta ^{2}$ are

$$\begin{aligned} &c=W\boldsymbol{\cdot } \frac{1}{2} ( \boldsymbol{\mu }_{1} + \boldsymbol{\mu }_{0} ) + b, \\ &\eta _{i} =W\boldsymbol{\cdot } \boldsymbol{\mu }_{i} +b-c, \\ &\zeta ^{2} = W^{T} \Sigma \mathrm{W}, \end{aligned}$$

with bias term *b*. The error rate from Eq. (6) then becomes

$$ \varepsilon = \frac{1}{2} \int _{-\infty }^{0} \frac{1}{\sqrt{2 \zeta ^{2} \pi }} e^{\frac{- ( w- \eta _{1} )^{2}}{2 \zeta ^{2}}} \,dw+ \frac{1}{2} \int _{0}^{\infty } \frac{1}{\sqrt{2 \zeta ^{2} \pi }} e^{\frac{- ( w- \eta _{0} )^{2}}{2 \zeta ^{2}}} \,dw. $$

Details of the full integration of error can be found in Section A.3. The final expression is

$$ \varepsilon = \frac{1}{2} \operatorname{erf}c \biggl( \frac{\eta _{1}}{\sqrt{2 \zeta ^{2}}} \biggr). $$

This expression is further simplified by introducing the squared Mahalanobis distance $d^{2}$

7 $$ \varepsilon = \frac{1}{2} \operatorname{erf}c \biggl( \frac{1}{2 \sqrt{2}} \sqrt{d^{2}} \biggr), $$

where

8 $$ d^{2} = \Delta \boldsymbol{\mu }^{T} \Sigma ^{-1} \Delta \boldsymbol{\mu }. $$

Because of equal class covariance, the above expression has the property that

$$ d ( \boldsymbol{\mu }_{0}, \boldsymbol{\mu }_{1} ) = d ( \boldsymbol{\mu }_{1},\boldsymbol{\mu }_{0} ) =d. $$

Using Eq. (8), we rewrite $d^{2}$ from the network parameters:

$$\begin{aligned} d^{2} &= \frac{1}{1- \rho ^{2}} \biggl[ \frac{1}{\sigma _{x}^{2}} \Delta \mu _{x}^{2} + \frac{1}{\sigma _{y}^{2}} \Delta \mu _{y}^{2} -2\rho \frac{1}{\sqrt{\sigma _{x}^{2} \sigma _{y}^{2}}} \Delta \mu _{x} \Delta \mu _{y} \biggr], \\ &= \frac{2}{1- \rho ^{2}} \biggl[ \frac{\tau _{x}}{\beta _{x}^{2} \alpha _{x}} \Delta \nu _{x}^{2} + \frac{\tau _{y}}{\beta _{y}^{2} \alpha _{y}} \Delta \nu _{y}^{2} -2 \rho \sqrt {\frac{\tau _{x}}{\beta _{x}^{2} \alpha _{x}} \frac{\tau _{y}}{\beta _{y}^{2} \alpha _{y}}} \Delta \nu _{x} \Delta \nu _{y} \biggr]. \end{aligned}$$

As the ratio $\Delta \mu _{u} / \sqrt{\sigma _{u}^{2}}$ appears often in the above solution, we simplify our notation by introducing

$$ r_{u} = \frac{\Delta u_{u}}{\sqrt{\sigma _{u}^{2}}} = \Delta \nu _{u} \sqrt {\frac{\tau _{u}}{\beta _{u}^{2} \alpha _{u}}}. $$

This expression simplifies the Mahalanobis distance to

$$ d^{2} = \frac{1}{1- \rho ^{2}} \bigl[ r_{x}^{2} + r_{y}^{2} -2\rho r_{x} r_{y} \bigr]. $$

The full derivation of expected error using Mahalanobis distance is found in Sections A.3–A.4. The above analysis provides a relationship between classification error and the network parameters of the population model. In the sections to follow, we explore the various links between these quantities.

## Noise correlation

Neurons that are in close physical proximity exhibit correlations in their activity. An extensive body of work has examined the impact of these noise correlations on behavioral tasks [26] and the activity of brain circuits [27–35]. Noise correlations may be advantageous or detrimental to cognitive and sensory processing; however, the specific network-level properties that give rise to these effects have not been fully elucidated.

In the proposed model, the effect of noise correlation on classification error is highly dependent upon the sensory inputs ($\nu _{1}$ and $\nu _{2}$). We distinguish four main cases that lead to qualitatively different conclusions on the impact of noise correlations. Details of these analyses are provided in Sections A.5–A.6.

**Figure 2:**
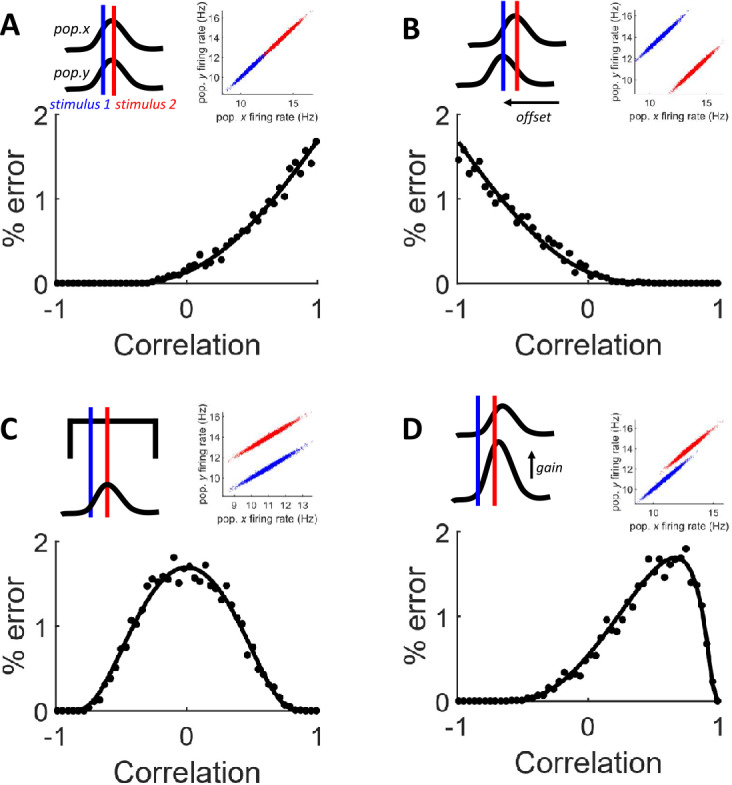
Impact of noise correlation on Fisher linear discriminant analysis. (**A**) Scenario where the tuning curves are the same for both populations of neurons, leading to $r_{x} \rightarrow r_{y}$. Top left: illustration of tuning curves (black lines) and stimulus orientations (blue and red lines). Top right: example of numerical responses to two stimuli (red and blue circles), with noise correlation of 0.9. Bottom: solid line, analytical estimate. Filled black circles, numerical simulations. (**B**) Scenario where the tuning curves are offset by a fixed orientation. In this case, $r_{x} \rightarrow $ -$r_{y}$. (**C**) Symmetrical case arising when one of the populations (for instance, *x*) generates the same firing rate for a range of stimulus orientations, leading to $r_{x} =0$. (**D**) Case where one population has higher gain, leading to $\vert r_{x} \vert \neq \vert r_{y} \vert $

A first case arises when the tuning curves of populations *x* and *y* are identical in terms of both their orientation preference and their gain (Fig. 2(A)). In this case, $r_{x} \rightarrow r_{y}$, leading to monotonically increasing error as a function of correlation. Intuitively, this happens because correlation forces the firing rate distributions to “stretch” towards each other. We verified the analytical solution by comparing it to numerical estimates of the error rate as a function of noise correlation. These numerical estimates were obtained with Eq. (1), where populations *x* and *y* both received inputs $\nu _{1} =11$ and $\nu _{2} =14$ in order for the model to mimick a scenario where the two populations have identical tuning properties. The goal here is not to capture the model’s response to a continuum of stimulus values along the tuning curves, but rather to illustrate the behavior of the model using discrete stimuli. We set $\tau =1$, $\beta =1$, and $\alpha =1$ for both populations. We then numerically generated 5000 points per stimulus class. A subset of 80% of the total number of data points were randomly selected to train the LDA classifier. The proportion of misclassified points was calculated based on the remaining data points. We found good agreement between the numerical estimates and analytical solution (Fig. 2(A)).

Note that the range of error may be increased by moving the firing rate distributions closer to each other without altering the overall shape of the function relating error and noise correlation. While the goal here was to show the distribution of readout error across a broad range of correlation values, we acknowledge that not all combinations of tuning curves and noise correlations are physiologically plausible. In fact, while noise correlations in cortex vary across experimental conditions, regions, and behavioral states, they are typically reported to be on the order of 0.1–0.3 for nearby cells [26]. Therefore, extreme values (both positive and negative) are unlikely to arise in living circuits.

In a second scenario, the two populations are offset in terms of their orientation preference (Fig. 2(B)). We examined classification error in this scenario by setting the input of population *x* to $\nu _{1} =11$ and $\nu _{2} =14$, while population *y* was set to $\nu _{1} =14$ and $\nu _{2} =11$. Analytically, this scenario leads to $r_{x} \rightarrow - r_{y}$, resulting in a monotonically decreasing error as correlation increases from −1 to 1. Intuitively, this scenario arises because correlation stretches the distributions of responses along parallel lines, decreasing the overlap between them.

A third case arises when the tuning curve of one of the two populations yields the same response for two stimuli (Fig. 2(C)). This happens if the tuning curve of population *x* exhibits a broad region where firing rate remains constant despite changes in stimulus orientation. Analytically, this would lead to $r_{x} =0$. We illustrate this scenario by setting $\nu _{1} =11$ and $\nu _{2} =11$ for population *x*, and $\nu _{1} =11$ and $\nu _{2} =14$ for population *y*. This case yields a “symmetrical” effect of correlation on readout error, where maximum error is found at $\rho _{*} =0$ and error tends towards zero as *ρ* approaches either 1 or −1.

Finally, a fourth scenario occurs when the two populations have tuning curves that are aligned in terms of orientation preference, but where one population has higher response gain (Fig. 2(D)). This case is defined by $\vert r_{x} \vert \neq \vert r_{y} \vert $. Error tends to zero as noise correlation (*ρ*) goes to either −1 or 1. The correlation associated with maximum error is found somewhere in between these extremes and is given by

9 $$ \rho _{*} = \frac{\min ( r_{x}^{2}, r_{y}^{2} )}{r_{x} r_{y}}. $$

To illustrate this scenario, we set $\nu _{1} =11$ and $\nu _{2} = $13 for population *x*, and $\nu _{1} =11$ and $\nu _{2} =14$ for population *y*. Graphically, this scenario arises when noise correlation “stretches” the distribution of responses along parallel lines and their centroids do not align on either dimension. Starting from a correlation of zero, as correlation increases, the distributions will stretch towards each other, thus increasing overlap and error. After a maximum overlap defined by $\rho _{*}$, further stretching of the distributions will force them to spread too thinly for them to overlap, until the extreme case of a correlation of one, where both distributions would appear as perfectly parallel lines, leading to zero error.

A continuum of cases exists between the different scenarios illustrated in Fig. 2(A)–(D). For instance, the peak error ($\rho _{*}$) in Fig. 2(D) can shift to lower correlation values by offsetting one of the tuning curves, yielding a curve closer to Fig. 2(B).

In sum, the above results show that, depending upon the structure of the input delivered to the two neural populations, noise correlations produce widely different effects on classification error. While insights into these results can be obtained without the full formalism described here [34], such formalism becomes pivotal when examining the effect of specific network parameters, as described next.

## Impact of noise gain on classification error

To explore the effect of network parameters on error, we first modify Eq. (9) as follows:

10 $$ \rho _{*} = \frac{\min ( r_{x}^{2}, r_{y}^{2} )}{r_{x} r_{y}} = \textstyle\begin{cases} \frac{r_{x}}{r_{y}} &\text{if }\vert r_{x} \vert < \vert r_{y} \vert , \\ \frac{r_{y}}{r_{x}}& \text{if }\vert r_{x} \vert > \vert r_{y} \vert , \end{cases} $$

where the ratio $r_{x} / r_{y}$ can be expressed using network parameters

$$ \frac{r_{x}}{r_{y}} = \frac{\Delta \nu _{x}}{\Delta \nu _{y}} \frac{\beta _{y} \sqrt{\tau _{x} \alpha _{y}}}{\beta _{x} \sqrt{\tau _{y} \alpha _{x}}}. $$

We define a set containing all network parameters $G_{u}=\{\alpha _{u}, \tau _{u}, \beta _{u}, \Delta \nu _{u}\}$. If *g* is a subset of these parameters, we can manipulate them using a function *f* (*g*) while setting the other parameters to a constant $c_{g}$. In this way, we can rewrite Eq. (10) as

$$ \rho _{*} = \textstyle\begin{cases} f ( g ) c_{g} &\text{if }\vert r_{x} \vert < \vert r_{y} \vert , \\ f(g )^{-1} c_{g}^{-1} &\text{if }\vert r_{x} \vert > \vert r_{y} \vert . \end{cases} $$

We can investigate the effect of network parameters on $\rho _{*} $. For example, the effect of noise gain ($\beta _{x}$ and $\beta _{y}$) on $\rho _{*}$ when keeping all other parameters constant except for the input is expressed as

$$ \rho _{*} =f ( \beta _{y}, \beta _{x} ) c_{\beta _{y}, \beta _{x}} = \frac{\beta _{y}}{\beta _{x}} \biggl( \frac{\Delta \nu _{x}}{\Delta \nu _{y}} \frac{\sqrt{\tau _{x} \alpha _{y}}}{\sqrt{\tau _{y} \alpha _{x}}} \biggr) $$

for $\vert r_{x} \vert < \vert r_{y} \vert $.

**Figure 3:**
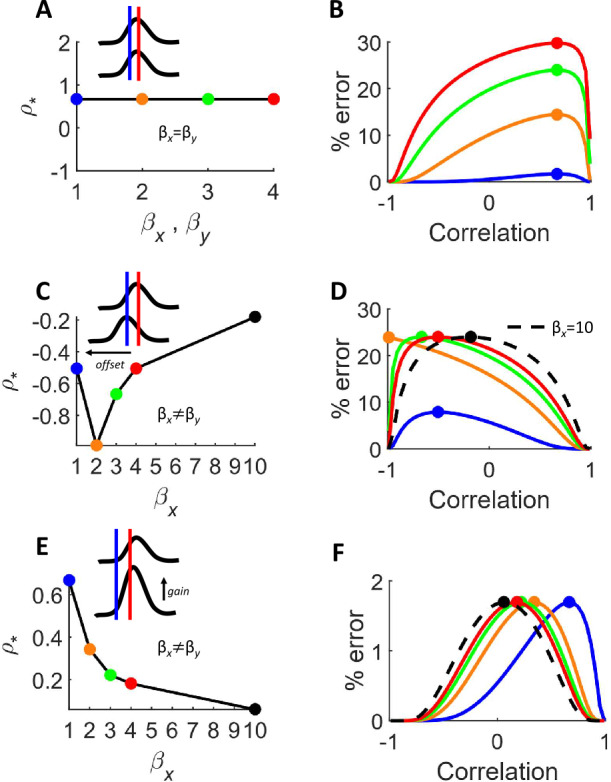
Influence of noise gain on discrimination error. (**A**) Scenario where the noise gains of both populations ($\beta _{x}$ and $\beta _{y}$) are adjusted simultaneously. In this case, the value of noise correlation leading to maximal error ($\rho _{*}$) remains constant. Inset: tuning curves for the two populations. (**B**) Error as a function of noise correlation for four different values of noise gain, with colors corresponding to the colored circles in panel “A”. Filled circles indicate $\rho _{*}$. (**C**) Impact of modifying the noise gain of population *x* only. (**D**) Different values of noise gain for population *x*. (**E**) Scenario taken from panel D of Fig. 2, showing a monotonic decrease in $\rho _{*}$ when increasing $\beta _{x}$. (**F**) Impact of $\beta _{x}$ on classification error

For illustration purposes, we explored the scenario described in Fig. 2(A), where two populations have equivalent tuning properties. Keeping all parameters constant while altering both $\beta _{x}$ and $\beta _{y}$ simultaneously has no effect on $\rho _{*}$ (Fig. 3(A)). The main impact is an increase in the amount of classification error (Fig. 3(B)). This result is not surprising: increasing the gain of the noise worsens readout performance.

However, markedly different results emerge in a scenario where tuning curves are offset (Fig. 2(B)) and $\beta _{x}$ is altered while keeping $\beta _{y}$ unchanged. In this case, $\rho _{*} =f ( \beta _{x} ) c_{\beta _{x}}$ with $c_{\beta _{x}}$ given by

$$ c_{\beta _{x}} = \frac{\beta _{y} \Delta \nu _{x} \sqrt{\tau _{x} \alpha _{y}}}{\Delta \nu _{y} \sqrt{\tau _{y} \alpha _{x}}}, $$

and $f ( \beta _{x} ) =1/ \beta _{x}$. Alterations in $\beta _{x}$ impact $\rho _{*}$ in a non-monotonic fashion (Fig. 3(C)). A small increase from $\beta _{x} =1$ to $\beta _{x} = $2 shifts $\rho _{*}$ towards a more negative value. However, further increasing to $\beta _{x} =3$ and $\beta _{x} =4$ increases $\rho _{*}$ and alters the relationship between correlation and readout error (Fig. 3(D)).

Hidden in these results is a counter-intuitive finding: under certain circumstances, increasing $\beta _{x}$ leads to a decrease in classification error. This can be seen with $\beta _{x} =10$ (Fig. 3(D), dashed line), leading to lower error than $\beta _{x} =3$ (green line) and $\beta _{x} =4$ (red line) for negative correlations. Intuitively, this can happen when increasing $\beta _{x}$ stretches the distribution of activity for population *x* along a single dimension away from the classification boundary [13]. Similar findings are borne out of graphical explanations where noise covariance stretches the distribution of firing rates [36].

The benefits of noise gain are even more pronounced in a scenario where one population has higher gain than the other, as in Fig. 2(D). In this case, $\beta _{x}$ monotonically shifts $\rho _{*}$ towards decreasing values (Fig. 3(E)). For a broad range of positive correlation values, a high noise gain ($\beta _{x} >1$) leads to lower classification error (Fig. 3(F)).

## Impact of dynamical parameters

The approach described in the previous section can be applied to study the impact of the model’s dynamical parameters on readout error. The two parameters of interest are the leak term (*α*) and the time constant (*τ*).

The effect of the time constants $\tau _{x}$ and $\tau _{y}$ on $\rho _{*}$ can be expressed as

$$ \rho _{*} = \frac{\tau _{x}}{\tau _{y}} \biggl( \frac{\Delta \nu _{x}}{\Delta \nu _{y}} \frac{\beta _{y} \sqrt{\alpha _{y}}}{\beta _{x} \sqrt{\alpha _{x}}} \biggr) $$

for $\vert r_{x} \vert < \vert r_{y} \vert $. To study the effect of a single term (e.g., $\tau _{x}$), we set $\rho _{*} =f ( \tau _{x} ) c_{ \tau _{x}}$ with $\mathrm{c}_{\tau _{x}}$ given by

$$ c_{\tau _{x}} = \frac{\beta _{y} \Delta \nu _{x} \sqrt{\alpha _{y}}}{\beta _{x} \Delta \nu _{y} \sqrt{\tau _{y} \alpha _{x}}}, $$

and $f ( \tau _{x} ) = \tau _{x}$. Similarly, the role of leak terms $\alpha _{x}$ and $\alpha _{y}$ on $\rho _{*}$ is

$$ \rho _{*} = \frac{\alpha _{y}}{\alpha _{x}} \biggl( \frac{\Delta \nu _{x}}{\Delta \nu _{y}} \frac{\beta _{y} \sqrt{\tau _{x}}}{\beta _{x} \sqrt{\tau _{y}}} \biggr). $$

**Figure 4:**
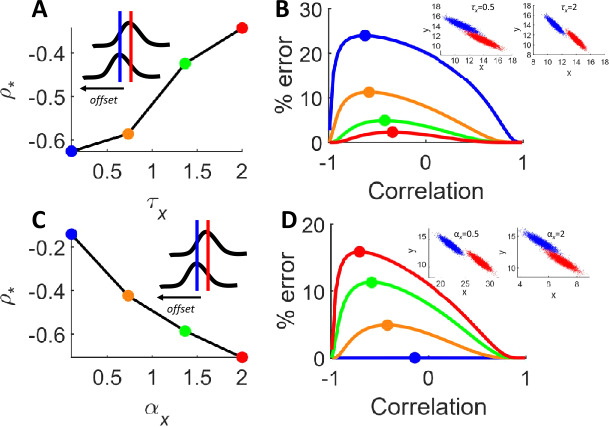
Mediating role of dynamical parameters. (**A**) In a scenario where tuning curves are offset by a fixed orientation, increasing the time constant $\tau _{x}$ leads to an increase in correlation associated with maximal error ($\rho _{*}$). (**B**) Different values of $\tau _{x}$ (colors corresponding to panel “A”) alter the relation between noise correlation and readout error. Inset: examples of firing rate distributions across two stimuli (shown in blue and red). (**C**) Increasing the leak term $\alpha _{x}$ leads to a decrease in $\rho _{*}$. (**D**) Readout error across different values of $\alpha _{x}$ (see panel “C” for colors). Inset: examples of firing rate distributions

For a single term ($\alpha _{x}$), we have $\rho _{*} =f ( \alpha _{x} ) c_{ \alpha _{x}}$ with

$$ c_{\alpha _{x}} = \frac{\beta _{y} \Delta \nu _{x} \sqrt{\tau _{x} \alpha _{y}}}{\beta _{x} \Delta \nu _{y} \sqrt{\tau _{y}}}, $$

and $f ( \alpha _{x} ) =1/ \alpha _{x}$. Taking one scenario as illustration, we examined the case where tuning curves are offset by a fixed orientation ($r_{x} \rightarrow $ -$r_{y}$). In this case, the time constant affects the relation between noise correlation and readout error, with larger values of $\tau _{x}$ shifting $\rho _{*}$ towards smaller negative values of correlation (Fig. 4(A)). The reason for this shift follows from an earlier example (Fig. 2(D)), where an increased correlation resulted in greater overlap between the firing rate distributions, but only up to a point beyond which these distributions became too narrow to overlap. With larger values of $\tau _{x}$, a given correlation does not create as much overlap as it would for smaller values of $\tau _{x}$, thus leading to a shift in $\rho _{*}$.

The overall impact of a larger time constant is a decrease in classification error (Fig. 4(B)): as $\tau _{x}$ increases, there is less overlap between the distributions of firing rate across stimuli (Fig. 4(B), inset). By contrast, shifting the leak term $\alpha _{x}$ towards higher values decreases $\rho _{*}$ (Fig. 4(C)) and increases overall readout error (Fig. 4(D)). The impact of increasing $\alpha _{x}$ on error is due to an increase in the overlap between firing rate distributions (Fig. 4(D), inset). The inverse effects of $\tau _{x}$ and $\alpha _{x}$ on these distributions explain their opposite impact on $\rho _{*}$.

**Figure 5:**
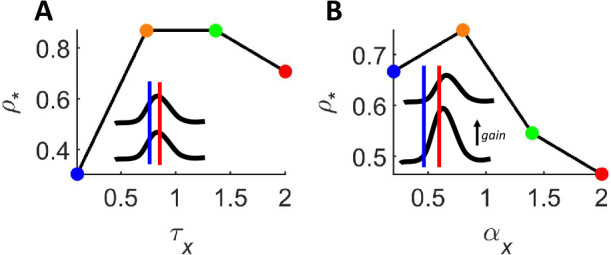
Nonlinear impact of dynamical parameters on classification error. (**A**) In a scenario where the tuning curves of the two neural populations are equivalent, $\tau _{x}$ and $\rho _{*}$ have a non-monotonic relation. (**B**) In a scenario where the gain of one tuning curve is larger, $\alpha _{x}$ and $\rho _{*}$ are non-monotonically related

More complex, non-monotonic relations between $\rho _{*}$ and values of $\tau _{x}$ and $\alpha _{x}$ are found in different scenarios where tuning curves of the two populations are aligned (Fig. 5(A)) or when the gain of one population is larger (Fig. 5(B)).

Together, these results show that the integration time constant and leak term of the population model mediate the impact of noise correlation on classification error by shifting the value $\rho _{*}$ at which correlation reaches maximal error. The impact of network parameters on readout error is therefore not straightforward to describe but is brought to light using a framework that derives error estimates from the dynamical parameters of a population model.

## Discussion

This work described an analytical framework for performing Fisher linear decoding in a rate-based neural model. With this formalism, we began by capturing well-documented findings on the role of noise gain and correlations on discrimination error. Going further, the framework allowed us to analytically examine the mediating role of dynamical parameters (neuronal leak and time constant) on the relation between noise correlation and error. Overall, this framework suggests that linear decoding is highly sensitive to dynamical model parameters as well as the characteristics of the sensory input.

One surprising finding was the presence of conditions where increased neuronal noise led to reduced classification error. This result was especially prominent when the gain of the two population tuning curves was unmatched (Fig. 3(E)–(F)). Taken together, our findings cover all possible qualitative scenarios where noise correlations have either a beneficial, detrimental, or null effect on decoding [36].

A related approach termed the leaky competing accumulator model was proposed in order to account for perceptual decision making [37]. Some key differences exist between this model and ours. Firstly, our framework assumes a steady-state of neural activity that is characteristic of a decision point and does not capture the time-course of deliberation. Our framework assumes an optimal bound on decision accuracy given a linear decoder, representing a ceiling in accuracy that would be associated with long response times (typically >500 ms in human subjects). Secondly, the accumulator model provides explicit connections, through lateral inhibition, that modulate correlations. These lateral connections, however, may also impact firing rates. By comparison, our framework isolates analytically the contribution of firing rates and correlations, and examines their relative role on perceptual discrimination.

It would be challenging to speculate on whether the analytical results provided would generalize to other classes of neural network models, particularly those that include a non-linear transfer function [38]. However, our work opens the door to such analyses by describing a framework for linking neuronal readout and dynamical modeling.

*Limitations and future work*. While the framework described here strived to cover all possible scenarios involving firing rates, noise correlations, and network parameters, it is important to emphasize that not all such scenarios are plausible from a physiological standpoint. In particular, the framework treats firing rates and noise correlations as independent contributors to decoding error and allows for implausible cases where increases in firing rate would lead to an increase, a decrease, or no impact on correlations. Interactions between stimulus and noise correlations are a crucial factor limiting the coding capacity of neural circuits [1, 23] and should be considered alongside the dynamical parameters discussed in this work.

Several future directions based on the proposed framework will be worth exploring. First, the assumption of equal class covariances in LDA is challenged by experimental work showing input-dependent neuronal variance [39]. This assumption could be relaxed by replacing LDA with quadratic discriminant analysis, albeit at the cost of a more complex solution when relating readout error to model parameters.

An extension of the current framework could consider the impact of pooling more than two neural populations, as well as more than two stimuli, when performing decoding. This extension would be helpful in examining the interactions between several populations of neurons, each with a unique tuning curve. Going further, one could examine decoding error at the limit of a large number of neurons with heterogeneous tuning curves that vary in both orientation preference and gain [2].

*Conclusion*. In summary, this work described a theoretical framework that merges Fisher linear decoding with a population model of sensory integration. This approach highlighted the role of correlation, neuronal noise, and network parameters, revealing a broad range of potential outcomes where different conditions generated either detrimental, beneficial, or null impacts on classification performance. These results motivate further developments in theoretical work that systematically link neural network models to optimal decoders in order to reveal the impact of key neurophysiological variables on sensory information processing.

## Data Availability

Data sharing not applicable to this article as no datasets were generated or analyzed during the current study.

## Appendix

### A.1 Solving the integrator model as a linear differential equation

To solve the integrator model, we began by dropping the unit indices to alleviate the notation:

$$\begin{aligned} &\tau \frac{dx}{dt} =-\alpha x+ \nu _{i} +\beta \xi ( t ) \\ &\quad\Leftrightarrow\quad \frac{dx}{dt} =- \frac{\alpha }{\tau } x+ \frac{\nu _{i} +\beta \xi ( t )}{\tau } \\ &\quad\Leftrightarrow\quad \frac{dx}{dt} + \frac{\alpha }{\tau } x= \frac{\nu _{i} +\beta \xi ( t )}{\tau } \\ &\quad\Leftrightarrow\quad \frac{dx}{dt} +p ( t ) x=r(t) \end{aligned}$$

with $p ( t ) =\alpha /\tau $ and $r ( t ) =(\nu +\beta \xi ( t ) )/\tau $. We defined

$$\begin{aligned} u ( t ) &= e^{\int p ( t ) \,dt} \\ &= e^{\int \frac{\alpha }{\tau } \,dt} \\ &= e^{\frac{\alpha }{\tau } t}. \end{aligned}$$

Then,

$$\begin{aligned} &u ( t ) \biggl( \frac{dx}{dt} +p ( t ) x \biggr) =u ( t ) r ( t ) \\ &\quad\Leftrightarrow\quad e^{\frac{\alpha }{\tau } t} \frac{dx}{dt} + e^{\frac{\alpha }{\tau } t} \frac{\alpha }{\tau } x= e^{\frac{\alpha }{\tau } t} r ( t ). \end{aligned}$$

Applying the chain rule,

$$\begin{aligned} &\frac{d}{dt} \bigl( u ( t ) x \bigr) =u ( t ) r(t) \\ &\quad\Leftrightarrow\quad d \bigl( u ( t ) x \bigr) =u ( t ) r ( t ) \,dt \\ &\quad\Leftrightarrow\quad \int _{o}^{t} \,d \bigl( u ( s ) x \bigr) = \int _{0}^{t} u ( s ) r ( s ) \,ds \\ &\quad\Leftrightarrow\quad u ( s ) x \vert _{0}^{t} = \int _{0}^{t} u ( s ) r ( s ) \,ds \\ &\quad\Leftrightarrow\quad u ( t ) x-u ( 0 ) x_{0} = \int _{0}^{t} u ( s ) r ( s ) \,ds \\ &\quad\Leftrightarrow\quad x= u(t)^{-1} \biggl( x_{0} + \int _{0}^{t} u ( s ) r ( s ) \,ds \biggr) \\ &\phantom{\frac{d}{dt} \bigl( u ( t ) x \bigr)}= e^{- \frac{\alpha }{\tau } t} \biggl( x_{0} + \int _{0}^{t} e^{\frac{\alpha }{\tau } s} \frac{\nu _{i} +\beta \xi (s)}{\tau } \,ds \biggr) \\ &\phantom{\frac{d}{dt} \bigl( u ( t ) x \bigr)}= e^{- \frac{\alpha }{\tau } t} \biggl( x_{0} + \frac{1}{\tau } \int _{0}^{t} e^{\frac{\alpha }{\tau } s} \bigl( \nu _{i} +\beta \xi (s) \bigr) \,ds \biggr) \\ &\phantom{\frac{d}{dt} \bigl( u ( t ) x \bigr)}= e^{- \frac{\alpha }{\tau } t} \biggl( x_{0} + \frac{1}{\tau } \int _{0}^{t} e^{\frac{\alpha }{\tau } s} \nu _{i} \,ds+ \frac{1}{\tau } \int _{o}^{t} e^{\frac{\alpha }{\tau } s} \beta \xi (s) \,ds \biggr) \\ &\phantom{\frac{d}{dt} \bigl( u ( t ) x \bigr)}= e^{- \frac{\alpha }{\tau } t} \biggl( x_{0} + \frac{1}{\tau } \frac{\tau \nu _{i}}{\alpha } e^{\frac{\alpha }{\tau } s} \vert _{0}^{t} + \frac{\beta }{\tau } \int _{o}^{t} e^{\frac{\alpha }{\tau } s} \xi ( s ) \,ds \biggr) \\ &\phantom{\frac{d}{dt} \bigl( u ( t ) x \bigr)}= e^{- \frac{\alpha }{\tau } t} \biggl( x_{0} + \frac{\nu _{i}}{\alpha } \bigl[ 1- e^{\frac{\alpha }{\tau } s} \bigr] + \frac{\beta }{\tau } \int _{o}^{t} e^{\frac{\alpha }{\tau } s} \xi ( s ) \,ds \biggr) \\ &\phantom{\frac{d}{dt} \bigl( u ( t ) x \bigr)}= x_{0} e^{- \frac{\alpha }{\tau } t} - \frac{\nu _{i}}{\alpha } \bigl[ 1- e^{- \frac{\alpha }{\tau } t} \bigr] + e^{- \frac{\alpha }{\tau } t} \frac{\beta }{\tau } \int _{o}^{t} e^{\frac{\alpha }{\tau } s} \xi ( s ) \,ds, \\ &x= \frac{\nu _{i}}{\alpha } + \biggl[ x_{0} - \frac{\nu _{i}}{\alpha } \biggr] e^{- \frac{\alpha }{\tau } t} + \frac{\beta }{\tau } \int _{o}^{t} e^{- \frac{\alpha }{\tau } (t-s)} \xi ( s ) \,ds. \end{aligned}$$

### A.2 Expected value and variance

We sought to find the expected mean and variance of the random variable *x* such that

$$ x=\mu + ( x_{o} -\mu ) e^{-\theta t} +\sigma \int _{0}^{t} e^{-\theta (t-s)} \,d B_{s}. $$

The expected mean is

$$\begin{aligned} E [ x ] &=E \biggl[ \mu + ( x_{0} -\mu ) e^{-\theta t} +\sigma \int _{0}^{t} e^{-\theta ( t-s )} \,d B_{s} \biggr] \\ &=E [ \mu ] +E \bigl[ ( x_{0} -\mu ) e^{-\theta t} \bigr] +E \biggl[ \sigma \int _{0}^{t} e^{-\theta ( t-s )} \,d B_{s} \biggr]. \end{aligned}$$

Given the zero-mean property of Ito integrals,

$$ E \biggl[ \sigma \int _{0}^{t} e^{-\theta ( t-s )} \,d B_{s} \biggr] =0, $$

we have

11 $$ E [ x ] =\mu + ( x_{0} -\mu ) e^{-\theta t}. $$

The expected variance is

$$\begin{aligned} \operatorname{var} ( x )& =\operatorname{var} \biggl( \mu + ( x_{0} - \mu ) e^{-\theta t} +\sigma \int _{0}^{t} e^{-\theta (t-s)} \,d B_{s} \biggr) \\ &= \sigma ^{2} \operatorname{var} \biggl( \int _{0}^{t} e^{-\theta ( t-s )} \,d B_{s} \biggr) \\ &= \sigma ^{2} \biggl( E \biggl[ \biggl( \int _{0}^{t} e^{-\theta ( t-s )} \,d B_{t} \biggr)^{2} \biggr] -E \biggl[ \int _{0}^{t} e^{-\theta ( t-s )} \,d B_{s} \biggr]^{2} \biggr) \\ &= \sigma ^{2} E \biggl[ \biggl( \int _{0}^{t} e^{-\theta ( t-s )} \,d B_{s} \biggr)^{2} \biggr]. \end{aligned}$$

By Ito isometry,

$$ \sigma ^{2} E \biggl[ \biggl( \int _{0}^{t} e^{-\theta ( t-s )} \,d B_{t} \biggr)^{2} \biggr] = \sigma ^{2} E \biggl[ \int _{0}^{t} \bigl( e^{-\theta ( t-s )} \bigr)^{2} \,ds \biggr]. $$

Hence, the expected variance can be concisely written as

$$\begin{aligned} \operatorname{var} ( x ) &= \sigma ^{2} \int _{0}^{t} e^{-2\theta ( t-s )} \,ds \\ &=\sigma ^{2} \biggl[ - \frac{e^{-2\theta ( t-s )}}{-2\theta } \biggr]_{0}^{t} \\ &= \frac{\sigma ^{2}}{2\theta } \bigl[ e^{-2\theta ( t-t )} e^{-2\theta ( t-0 )} \bigr] \\ &= \frac{\sigma ^{2}}{2\theta } \bigl[ 1- e^{-2\theta t} \bigr]. \end{aligned}$$

### A.3 Classification error

The classification error as a function of neural activity is given by

$$\begin{aligned} \varepsilon &= \frac{1}{2} \int _{-\infty }^{0} \frac{1}{\sqrt{2 \zeta ^{2} \pi }} e^{\frac{- (w- \eta _{1} )^{2}}{2 \zeta ^{2}}} \,dw+ \frac{1}{2} \int _{0}^{\infty } \frac{1}{\sqrt{2 \zeta ^{2} \pi }} e^{\frac{- (w- \eta _{0} )^{2}}{2 \zeta ^{2}}} \,dw \\ &= \frac{1}{2} \frac{1}{\sqrt{2 \zeta ^{2} \pi }} \biggl[ \sqrt{\frac{\pi \zeta ^{2}}{2}} \operatorname{erf}c \biggl( \frac{\eta _{1}}{\sqrt{2 \zeta ^{2}}} \biggr) + \sqrt{\frac{\pi }{2}} \biggl( \sqrt{\zeta ^{2}} \operatorname{erf} \biggl( \frac{\eta _{0}}{\sqrt{2 \zeta ^{2}}} \biggr) + \sqrt{ \zeta ^{2}} \biggr) \biggr] \\ &= \frac{1}{2} \frac{1}{\sqrt{2 \zeta ^{2} \pi }} \sqrt{\frac{\pi \zeta ^{2}}{2}} \biggl[ \operatorname{erf}c \biggl( \frac{\eta _{1}}{\sqrt{2 \zeta ^{2}}} \biggr) +\operatorname{erf} \biggl( \frac{\eta _{0}}{\sqrt{2 \zeta ^{2}}} \biggr) +1 \biggr] \\ &= \frac{1}{4} \biggl[ 1-\operatorname{erf} \biggl( \frac{\eta _{1}}{\sqrt{2 \zeta ^{2}}} \biggr) +\operatorname{erf} \biggl( \frac{\eta _{0}}{\sqrt{2 \zeta ^{2}}} \biggr) +1 \biggr] \\ &= \frac{1}{4} \biggl[ 2-\operatorname{erf} \biggl( \frac{\eta _{1}}{\sqrt{2 \zeta ^{2}}} \biggr) +\operatorname{erf} \biggl( \frac{-\eta _{1}}{\sqrt{2 \zeta ^{2}}} \biggr) \biggr] \\ &= \frac{1}{2} \biggl[ 1-\operatorname{erf} \biggl( \frac{\eta _{1}}{\sqrt{2 \zeta ^{2}}} \biggr) \biggr] \\ &= \frac{1}{2} \operatorname{erf}c \biggl( \frac{\eta _{1}}{\sqrt{2 \zeta ^{2}}} \biggr). \end{aligned}$$

Substituting the mean and variance from the previous section, this becomes

$$\begin{aligned} \varepsilon &= \frac{1}{2} \operatorname{erf}c \biggl( 2 \frac{d^{2}}{4 \sqrt{2 \,d^{2}}} \biggr) \\ &= \frac{1}{2} \operatorname{erf}c \biggl( \frac{1}{2 \sqrt{2}} \sqrt{d^{2}} \biggr). \end{aligned}$$

### A.4 Mahalanobis distance

We began with the following definitions:

$$\begin{aligned} &W= (2 \Sigma )^{-1} \Delta \boldsymbol{\mu }, \\ &c=W\boldsymbol{\cdot } \frac{1}{2} ( \boldsymbol{\mu }_{1} + \boldsymbol{\mu }_{0} ) +b, \\ &\eta _{i} =W\boldsymbol{\cdot } \boldsymbol{\mu }_{i} + b-c, \\ &\varsigma ^{2} = W^{T} \Sigma \mathrm{W}. \end{aligned}$$

Expanding $\eta _{i}$ yields

$$\begin{aligned} \eta _{i} &=W\boldsymbol{\cdot } \boldsymbol{\mu }_{i} + b- \biggl( W\boldsymbol{\cdot } \frac{1}{2} ( \boldsymbol{\mu }_{1} + \boldsymbol{\mu }_{0} ) + b \biggr) \\ &=W\boldsymbol{\cdot } \biggl( \boldsymbol{\mu }_{i} - \frac{1}{2} ( \boldsymbol{\mu }_{1} + \boldsymbol{\mu }_{0} ) \biggr). \end{aligned}$$

Given

$$\begin{aligned} \eta _{1} &= \frac{1}{2} W\boldsymbol{\cdot } ( \boldsymbol{\mu }_{1} + \boldsymbol{\mu }_{0} ) \\ &= \frac{1}{2} W\boldsymbol{\cdot } \Delta \boldsymbol{\mu } \\ &=- \eta _{0}, \end{aligned}$$

we expanded *W* using the property $u\boldsymbol{\cdot }\upsilon = u^{T} \upsilon $,

$$\begin{aligned} \eta _{1} &= \frac{1}{2} \bigl[ (2 \Sigma )^{-1} \Delta \boldsymbol{\mu } \bigr] \boldsymbol{\cdot } \Delta \boldsymbol{\mu } \\ &= \frac{1}{4} \bigl[ \Sigma ^{-1} \Delta \boldsymbol{\mu } \bigr]^{T} \Delta \boldsymbol{\mu } \\ &= \frac{1}{4} \Delta \boldsymbol{\mu }^{T} \bigl( \Sigma ^{-1} \bigr)^{T} \Delta \boldsymbol{\mu } \\ &= \frac{1}{4} \Delta \boldsymbol{\mu }^{T} \Sigma ^{-1} \Delta \boldsymbol{\mu }. \end{aligned}$$

Hence, the squared Mahalanobis distance between means is

$$ d^{2} = \Delta \boldsymbol{\mu }^{T} \Sigma ^{-1} \Delta \boldsymbol{\mu }. $$

We can rewrite $\eta _{i}$ as

$$ \eta _{1} = \frac{1}{4} d^{2} =- \eta _{0}. $$

Similarly, for the variance $\varsigma ^{2}$,

$$\begin{aligned} \varsigma ^{2} &= \bigl[ (2 \Sigma )^{-1} \Delta \boldsymbol{ \mu } \bigr]^{T} \Sigma (2\Sigma )^{-1} \Delta \boldsymbol{\mu } \\ &= \frac{1}{4} \Delta \boldsymbol{\mu }^{T} \Sigma ^{-1} \Sigma \Sigma ^{-1} \Delta \boldsymbol{\mu } \\ &= \frac{1}{4} \Delta \boldsymbol{\mu }^{T} \Sigma ^{-1} \Delta \boldsymbol{\mu } \\ &= \frac{1}{4} d^{2}. \end{aligned}$$

### A.5 Derivation of error

We analyzed the extrema of the error function in relation to noise correlation by taking its first derivative through the chain rule

12 $$\begin{aligned} \frac{d\varepsilon }{d\rho }& = \frac{d}{d\rho } \biggl( \frac{1}{2} \operatorname{erf}c \biggl( \frac{1}{2 \sqrt{2}} \sqrt{d^{2}} \biggr) \biggr) \\ &= \frac{1}{2} \frac{d}{d\rho } \bigl( \operatorname{erf}c ( z ) \bigr) \\ &= \frac{1}{2} \frac{d}{dz} \bigl( \operatorname{erf}c ( z ) \bigr) \frac{dz}{d d^{2}} \frac{d d^{2}}{d\rho }, \end{aligned}$$

with

13 $$ d^{2} = \frac{1}{1- \rho ^{2}} \bigl[ r_{x}^{2} + r_{y}^{2} -2\rho r_{x} r_{y} \bigr]. $$

The first derivative is given by

14 $$\begin{aligned} \frac{d}{dz} \bigl( \operatorname{erf}c ( z ) \bigr) &= \frac{-2 e^{- z^{2}}}{\sqrt{\pi }} \\ &= \frac{-2 e^{- ( \frac{1}{2 \sqrt{2}} \sqrt{d^{2}} )^{2}}}{\sqrt{\pi }} \\ &= \frac{-2 e^{- \frac{1}{8} d^{2}}}{\sqrt{\pi }}. \end{aligned}$$

The second derivative is

15 $$\begin{aligned} \frac{dz}{d d^{2}} &= \frac{d}{d d^{2}} \frac{1}{2 \sqrt{2}} \sqrt{d^{2}} \\ &= \frac{1}{4 \sqrt{2 d^{2}}}. \end{aligned}$$

The third derivative is

16 $$\begin{aligned} \frac{d d^{2}}{d\rho } &= \frac{d}{d\rho } \biggl( \frac{1}{1- \rho ^{2}} \bigl[ r_{x}^{2} + r_{y}^{2} -2\rho r_{x} r_{y} \bigr] \biggr) \\ &= \bigl[ r_{x}^{2} + r_{y}^{2} -2\rho r_{x} r_{y} \bigr] \frac{d}{d\rho } \frac{1}{1- \rho ^{2}} + \frac{1}{1- \rho ^{2}} \frac{d}{d\rho } \bigl[ r_{x}^{2} + r_{y}^{2} -2\rho r_{x} r_{y} \bigr] \\ &= \bigl[ r_{x}^{2} + r_{y}^{2} -2\rho r_{x} r_{y} \bigr] \frac{2\rho }{(1- \rho ^{2} )^{2}} + \frac{1}{1- \rho ^{2}} [ -2 r_{x} r_{y} ] \\ &= \frac{1}{(1- \rho ^{2} )^{2}} \bigl[ \bigl(r_{x}^{2} + r_{y}^{2} -2\rho r_{x} r_{y} \bigr)2 \rho +\bigl(1- \rho ^{2} \bigr) (-2 r_{x} r_{y} ) \bigr] \\ &= \frac{1}{(1- \rho ^{2} )^{2}} \bigl[ \bigl(2\rho r_{x}^{2} + 2\rho r_{y}^{2} -2\rho 2\rho r_{x} r_{y} \bigr)+\bigl(-2 r_{x} r_{y} +2 r_{x} r_{y} \rho ^{2} \bigr) \bigr] \\ &= \frac{-2}{(1- \rho ^{2} )^{2}} \bigl[ \rho ^{2} r_{x} r_{y} - \rho \bigl( r_{x}^{2} +r_{y}^{2} \bigr)+ r_{x} r_{y} \bigr]. \end{aligned}$$

### A.6 Extrema of error

We evaluated the extrema of error by finding the points where Eqs. (14)–(16) are equal to zero,

17 $$\begin{aligned} \begin{aligned} &0= \frac{d}{dz} \bigl( \operatorname{erf}c ( z ) \bigr) \quad\Leftrightarrow\quad 0= \frac{-2 e^{- \frac{1}{8} d^{2}}}{\sqrt{\pi }}, \\ &d^{2} \rightarrow \infty. \end{aligned} \end{aligned}$$

We assumed that the ratios $r_{x}$ and $r_{y}$ are finite and the Euclidean distance between the distribution means is finite and non-null. In other words, if $d^{2} \rightarrow \infty $ it is exclusively due to the correlation coefficient. Then,

18 $$ d^{2} \rightarrow \infty \quad\Leftrightarrow\quad \vert \rho \vert \rightarrow 1. $$

We proceeded in a similar fashion for the second derivative (Eq. (15)):

19 $$\begin{aligned} \begin{aligned} &0= \frac{dz}{d d^{2}}\quad \Leftrightarrow\quad 0= \frac{1}{4 \sqrt{2 d^{2}}} \\ &d^{2} \rightarrow \infty \quad\Leftrightarrow\quad \vert \rho \vert \rightarrow 1. \end{aligned} \end{aligned}$$

The third derivative (Eq. (16)) is

20 $$\begin{aligned} \begin{aligned} 0= \frac{d d^{2}}{d\rho}\quad &\Leftrightarrow\quad 0= \frac{-2}{(1- \rho ^{2} )^{2}} \bigl[ \rho ^{2} r_{x} r_{y} -\rho \bigl( r_{x}^{2} + r_{y}^{2} \bigr) + r_{x} r_{y} \bigr], \\ &\Leftrightarrow \quad 0= \rho ^{2} r_{x} r_{y} -\rho \bigl( r_{x}^{2} + r_{y}^{2} \bigr) + r_{x} r_{y}. \end{aligned} \end{aligned}$$

Depending on network parameters, two cases are possible. One case arises if one of the ratios, either $r_{x}$ or $r_{y}$, is zero. This happens if the mean activity of one population is equal across inputs. If the mean activity of both units remained unchanged, the resulting multivariate distributions would overlap, thus breaking the basic assumptions justifying the choice of LDA. In this first case,

21 $$ 0= \frac{d d^{2}}{d\rho } \quad\Leftarrow\quad 0=\rho\quad \text{if }r_{x} =0 \text{ or } r_{y} =0. $$

The second case occurs when neither $r_{x}$ nor $r_{y}$ is zero:

$$\begin{aligned} &0= \frac{dd^{2}}{d\rho } \quad\Leftarrow\quad 0= \rho ^{2} -\rho \frac{r_{x}^{2} + r_{y}^{2}}{r_{x} r_{y}} +1 \\ &\quad\Leftrightarrow\quad \rho = \frac{\frac{r_{x}^{2} + r_{y}^{2}}{r_{x} r_{y}} \pm \sqrt{\frac{( r_{x}^{2} + r_{y}^{2} )^{2}}{r_{x}^{2} r_{y}^{2}} -4}}{2} \\ &\phantom{0}= \frac{r_{x}^{2} + r_{y}^{2} \pm \sqrt{( r_{x}^{2} + r_{y}^{2} )^{2} -4 r_{x}^{2} r_{y}^{2}}}{2 r_{x} r_{y}} \\ &\phantom{0}= \frac{r_{x}^{2} + r_{y}^{2} \pm \sqrt{r_{x}^{4} + r_{y}^{4} -2 r_{x}^{2} r_{y}^{2}}}{2 r_{x} r_{y}} \\ &\phantom{0}= \frac{r_{x}^{2} + r_{y}^{2} \pm \sqrt{(r_{x}^{2} - r_{y}^{2} )^{2}}}{2 r_{x} r_{y}} \\ &\phantom{0}= \frac{r_{x}^{2} + r_{y}^{2} \pm \vert r_{x}^{2} - r_{y}^{2} \vert }{2 r_{x} r_{y}} \\ &\phantom{0}= \frac{r_{x}^{2} + r_{y}^{2} \pm [ \max ( r_{x}^{2}, r_{y}^{2} ) - \min ( r_{x}^{2}, r_{y}^{2} ) ]}{2 r_{x} r_{y}}. \end{aligned}$$

The last expression can be decomposed into four distinct cases. First, when $r_{x}\rightarrow r_{y}$,

22 $$ \rho \rightarrow \frac{r_{y}^{2} + r_{y}^{2}}{2 r_{y} r_{y}} \rightarrow 1. $$

Second, when $r_{x} \rightarrow - r_{y}$,

23 $$ \rho \rightarrow \frac{r_{y}^{2} + r_{y}^{2}}{-2 r_{y} r_{y}} \rightarrow -1. $$

Third, when $r_{x} \neq r_{y}$, we examined the positive and negative roots of *ρ*. The positive root is

24 $$\begin{aligned} \rho _{+} &= \frac{r_{x}^{2} + r_{y}^{2} + \max ( r_{x}^{2}, r_{y}^{2} ) - \min ( r_{x}^{2}, r_{y}^{2} )}{2 r_{x} r_{y}} \\ &= \frac{\max (r_{x}^{2}, r_{y}^{2} )}{r_{x} r_{y}}. \end{aligned}$$

Because $\vert \max (r_{x}^{2}, r_{y}^{2} ) \vert >\vert r_{x} r_{y} \vert $ from the assumption that one ratio is smaller than the other (or unequal, non-null), this means that $\vert \rho _{+} \vert >1\ \forall r_{x}, r_{y}$. Since the correlation is bound in the range $\mathopen{]}-1,1[$, the positive root must be rejected. The negative root does not suffer from the same problem,

25 $$\begin{aligned} \rho _{-}& = \frac{r_{x}^{2} + r_{y}^{2} - \max ( r_{x}^{2}, r_{y}^{2} ) + \min ( r_{x}^{2}, r_{y}^{2} )}{2 r_{x} r_{y}} \\ &= \frac{\min ( r_{x}^{2}, r_{y}^{2} )}{r_{x} r_{y}}. \end{aligned}$$

Fourth, when either $r_{x} =0$ or $r_{y} =0$, $\rho =0$.

### A.7 Minima and maxima

We determined upward and downward trends of the error curve by calculating the sign of the derivative between the potential maxima (considering that they are mutually exclusive). Taking Eqs. (14)–(16) and substituting into Eq. (12),

26 $$\begin{aligned} &\frac{d\varepsilon }{d\rho } = \frac{1}{2} \frac{d}{dz} \bigl( \operatorname{erf}c ( z ) \bigr) \frac{dz}{d d^{2}} \frac{d d^{2}}{d\rho } \\ &\phantom{\frac{d\varepsilon }{d\rho }}= \frac{1}{2} \frac{-2 e^{- \frac{1}{8} d^{2}}}{\sqrt{\pi }} \frac{1}{4 \sqrt{2 d^{2}}} \frac{-2}{(1- \rho ^{2} )^{2}} \bigl[ \rho ^{2} r_{x} r_{y} -\rho \bigl( r_{x}^{2} + r_{y}^{2} \bigr) + r_{x} r_{y} \bigr] \\ &\quad\Leftrightarrow\quad \operatorname{sign} \biggl( \frac{d\varepsilon }{d\rho } \biggr) = \operatorname{sign} \bigl( \rho ^{2} r_{x} r_{y} - \rho \bigl( r_{x}^{2} + r_{y}^{2} \bigr) + r_{x} r_{y} \bigr). \end{aligned}$$

For the condition where $r_{x} \rightarrow r_{y}$,

27 $$\begin{aligned} \operatorname{sign} \biggl( \frac{d\varepsilon }{d\rho } \biggr)& =\operatorname{sign} \bigl( -\rho r_{y}^{2} \bigr) \\ &=-\operatorname{sign} ( \rho ). \end{aligned}$$

For the condition where either $r_{x} =0$ or $r_{y} =0$, we have already found the zeros of $\rho ^{2} r_{x} r_{y} -\rho ( r_{x}^{2} + r_{y}^{2} ) + r_{x} r_{y}$ to be $\rho _{-}$ and $\rho _{+}$. To determine $\operatorname{sign} ( d\varepsilon /d\rho )$, we need to know whether the extremum of the parabola is a minimum or a maximum,

$$ \frac{d^{2}}{d \rho ^{2}} \bigl( \rho ^{2} r_{x} r_{y} -\rho \bigl( r_{x}^{2} + r_{y}^{2} \bigr) + r_{x} r_{y} \bigr) =2>0. $$

Given $\rho _{+} >1$,

28 $$\begin{aligned} & \frac{d\varepsilon }{d\rho } >0 \quad\Leftrightarrow \quad p\in [ -1, \rho _{-} ] \end{aligned}$$

29 $$\begin{aligned} & \frac{d\varepsilon }{d\rho } < 0 \quad\Leftrightarrow\quad p\in [ \rho _{-},1 ]. \end{aligned}$$

Regardless of the conditions for $r_{x}$ and $r_{y}$, following Eqs. (27)–(29), the error curve as a function of correlation increases from $\rho =-1$ until its maximum, found at a value of $\rho _{*} =0$ or $\rho _{*} = \rho _{-}$, and then decreases until $\rho =1$.

## Abbreviations

LDA: Linear discriminant analysis
